# Does Empagliflozin Modulate Leukocyte–Endothelium Interactions, Oxidative Stress, and Inflammation in Type 2 Diabetes?

**DOI:** 10.3390/antiox10081228

**Published:** 2021-07-30

**Authors:** Francisco Canet, Francesca Iannantuoni, Aránzazu Martínez de Marañon, Pedro Díaz-Pozo, Sandra López-Domènech, Teresa Vezza, Blanca Navarro, Eva Solá, Rosa Falcón, Celia Bañuls, Carlos Morillas, Milagros Rocha, Víctor M. Víctor

**Affiliations:** 1 Service of Endocrinology and Nutrition, University Hospital Doctor Peset, Foundation for the Promotion of Health and Biomedical Research in the Valencian Region (FISABIO), 46017 Valencia, Spain; francasu@alumni.uv.es (F.C.); franian@alumni.uv.es (F.I.); amardema@alumni.uv.es (A.M.d.M.); pediazpo@alumni.uv.es (P.D.-P.); sandra.lopez@uv.es (S.L.-D.); vezza_ter@gva.es (T.V.); eva.sola@uv.es (E.S.); falcon_ros@gva.es (R.F.); carlos.morillas@uv.es (C.M.); 2 Department of Physiology, University of Valencia, 46010 Valencia, Spain; blanca.navarro-cubells@uv.es; 3 CIBERehd—Department of Pharmacology, University of Valencia, 46010 Valencia, Spain

**Keywords:** cardiovascular risk, empagliflozin, inflammation, oxidative stress, type 2 diabetes

## Abstract

Sodium-glucose co-transporter 2 inhibitors (iSGLT2) have been linked to cardiovascular risk reduction in patients with type 2 diabetes (T2D). However, their underlying molecular mechanisms remain unclear. This study aimed to evaluate the effects of empagliflozin, a novel potent and selective iSGLT-2, on anthropometric and endocrine parameters, leukocyte–endothelium interactions, adhesion molecules, ROS production, and NFkB-p65 transcription factor expression. According to standard clinical protocols, sixteen T2D patients receiving 10 mg/day of empagliflozin were followed-up for 24 weeks. Anthropometric and analytical measurements were performed at baseline, 12 weeks, and 24 weeks. Interactions between polymorphonuclear leukocytes and human umbilical vein endothelial cells (HUVECs), serum levels of adhesion molecules (P-Selectin, VCAM-1 and ICAM-1) and pro-inflammatory cytokines (TNF-α, IL-1β and IL-6), mitochondrial ROS levels, antioxidant enzymes (*SOD1* and *GPX1*), and NFkB-p65 were measured. We observed a decrease in body weight, BMI, and HbA1C levels from 12 weeks of treatment, which became more pronounced at 24 weeks and was accompanied by a significant reduction in waist circumference and glucose. Leukocyte–endothelium interactions were reduced due to an enhancement in the leukocyte rolling velocity from 12 weeks onwards, together with a significant decrease in leukocyte rolling flux and adhesion at 24 weeks. Accordingly, a significant decrease in ICAM-1 levels, mitochondrial ROS levels, and IL-6 and NFkB-p65 expression was observed, as well as an increase in *SOD1*. This pilot study provides evidence of the anti-inflammatory and antioxidant properties of empagliflozin treatment in humans, properties which may underlie its beneficial cardiovascular effects.

## 1. Introduction

Cardiovascular diseases (CVDs) are the most common cause of mortality in type 2 diabetic (T2D) patients, and hyperglycemia, hypertension, dyslipidemia, and obesity are important risk factors for CVDs. In particular, under chronic hyperglycemic conditions, elevated levels of circulating advanced glycation end products (AGEs) play a central role in the pathogenesis of the micro- and macrovascular complications related to T2D [1], promoting cellular dysfunction and regulating endothelial cell permeability, monocyte migration, and expression of adhesion molecules [2]. Another important aspect in the development of CVDs is the atherosclerotic process, which is mediated by peripheral polymorphonuclear leukocytes (PMNs). PMNs are activated under chronic hyperglycemia and play a crucial role in CVDs by promoting cellular and endothelial impairment due to vessel recruitment and leukocyte aggregation [3] or through reactive oxygen species (ROS) production, which reduces antioxidant defense systems such as superoxide dismutase 1 (SOD1) and glutathione peroxidase 1 (GPX1), thus leading to oxidative stress. Consequently, these events promote NF-κB activation, altering, in turn, pro-inflammatory gene expression and eventually inducing cardiovascular impairment [4].

In addition, the relationship between glycated hemoglobin levels (HbA1C), inflammation, and CVDs points to modulation of HbA1C levels as a potentially interesting therapeutic goal. In this sense, inhibitors of sodium and glucose co-transporter 2 (iSGLT2) are one of many classes of anti-diabetic agents and could represent an effective therapeutic strategy given their safety and potential both as a monotherapy and in combination with other anti-diabetic drugs [5]. Empagliflozin is an iSGLT2 approved for the treatment of adults with T2D and, as demonstrated by the EMPA-REG OUTCOME study, exerts both cardioprotective and renoprotective effects [6].

In the present pilot study, we investigated the potential therapeutic benefits of empagliflozin treatment (12 and 24 weeks) on leukocyte–endothelial interactions, adhesion molecules, mitochondrial ROS production, and NFkB-p65 expression, all of which are implicated in the development of atherosclerosis and CVDs.

## 2. Materials and Methods

### 2.1. Patients and Sample Collection

This is an observational and prospective follow-up study of a cohort of eighteen patients diagnosed with T2D according to the American Diabetes Association’s criteria and attending the Endocrinology Department of the University Hospital Doctor Peset (Valencia, Spain). Patients were recruited when physicians added empagliflozin to their usual treatment according to the hospital’s standard clinical protocols. Two of the eighteen patients were excluded from the study due to a lack of treatment adherence. Subjects were asked to follow a scheduled visit program that included follow-up at 12 and 24 weeks after the first visit.

The inclusion criteria were as follows: age between 40 and 70 years and evolution of diabetes greater than 10 years. The exclusion criteria were as follows: severe diabetic neuropathy, significant renal impairment (creatinine > 1.5 mg/dL or eGFR < 60 mL/min/1.73 m^2^), morbid obesity (BMI > 40 kg/m^2^), smoking habit or frequent alcohol intake, and chronic diseases other than those directly related to cardiovascular risk. Empagliflozin was administered orally at doses of 10 mg/day according to the normal clinical practice [6]. Measurements were assessed at baseline and at 12 and 24 weeks of empagliflozin treatment. The most common side effects for iSGLT2 treatment described in the literature are genital and urinary tract infections, but none of our subjects developed any of these conditions during the study. All subjects were informed about the study procedures and gave their informed written consent. The study was performed in compliance with the statement of ethical principles for medical research of the Declaration of Helsinki and obtained approval from the hospital’s ethics committee (CEIC 98/19).

### 2.2. Anthropometric and Biochemical Analysis

During the first and follow-up appointments, patients underwent a physical examination to determine the following anthropometrical parameters: weight (kg), height (m), waist circumference (cm), and systolic (SBP) and diastolic blood pressures (DBP, mmHg). After 12 h of overnight fasting, blood samples were taken from 8:00 to 10:00 a.m. and centrifuged (1.500 *g*, 10 min, 4 °C) to separate serum or plasma prior to determining biochemical and molecular parameters. Biochemical determinations were carried out by our hospital´s Clinical Analysis Service and evaluated as usual: glucose, triglycerides, and total cholesterol levels in serum were measured by means of an enzymatic method; insulin levels were calculated by immunochemiluminescence and insulin resistance was measured by homeostasis model assessment (HOMA-IR = [fasting insulin (μU/mL) × fasting glucose (mg/dL)]/405); percentage of HbA1C was measured with an automated glycohemoglobin analyzer (Arkray Inc., Kyoto, Japan); levels of high-density lipoprotein cholesterol (HDL-c) were assessed with a Beckman LX-20 autoanalyzer (Beckman Coulter, La Brea, CA, USA); low-density lipoprotein cholesterol (LDL-c) was estimated with Friedewald’s formula; and high-sensitive C-reactive protein (hs-CRP) levels were determined by an immunonephelometric assay (Behring Nephelometer II, Newark, DE, USA).

### 2.3. Leukocyte Isolation

Citrated blood samples were incubated for 45 min with 3% *w*/*v* dextran in phosphate-buffered saline solution (PBS; Sigma Aldrich, St. Louis, MO, USA). To isolate PMNs, supernatants were placed on Ficoll-Hypaque (GE Healthcare, Barcelona, Spain), and gradient centrifugation was performed (650g for 25 min at RT). The supernatant was discarded, and the bottom phase containing the PMN pellet was incubated for 5 min at RT with lysis buffer to eliminate the remaining erythrocytes. The sample was then centrifuged (1.200 rpm, 5 min) and washed twice with Hank´s Balance Salt Solution (HBSS; Sigma Aldrich, St. Louis, MO, USA). Finally, the pellet was resuspended in complete RPMI medium (Biowest-bw, Nuaillé, France) supplemented with 10% FBS. Aliquots of 1.0 × 10^6^ cell/mL were employed in the subsequent experiments.

### 2.4. Leukocyte–Endothelium Interactions, Pro-Inflammatory Cytokines, and Cellular Adhesion Molecule Evaluation

For adhesion assays, we used an ex vivo model based on a parallel plate flow chamber, as described before [7]. In brief, human umbilical vein endothelial cells (HUVECs) were harvested from fresh umbilical cords obtained from healthy donors. Primary cultures of HUVECs were grown over fibronectin-coated cell culture dishes (Corning, NY, USA) and incubated with complete Endothelial Cell Basal Medium-2 supplemented with Growth Medium-2 Supplement kit (both from PromoCell GmbH, Heidelberg, Germany) until HUVECs reached confluence. A portion of 5 × 25 mm of the HUVEC monolayer was exposed to the PMN flux and recorded using an inverted microscope (Nikon Eclipse TE 2000-S, Amstelveen, The Netherlands) coupled to a video camera (Sony Exware HAD, Koeln, Germany). Along the HUVEC monolayer, suspensions of PMNs were perfused at a flow rate of 0.36 mL/min (human blood flow rate in physiological condition). Real-time images of the flow-exposed monolayer were recorded for 5 min and further analyzed to extrapolate leukocyte rolling flux, rolling velocity, and adhesion [8].

Levels of pro-inflammatory markers (TNF-α, IL-1β, and IL-6) and cellular adhesion molecules in serum samples (P-Selectin, ICAM-1, and VCAM-1) were evaluated using a Luminex 200 flow analyzer system (Luminex Corp., Austin, TX, USA). Milliplex^®^ MAP human high sensitivity T Cell and Human Cardiovascular Disease Magnetic Bead Panel were purchased from Millipore Corporation (Billerica, MA, USA). The intra-serial CV was <5.0%, and the inter-serial CV was <15.0%, for all determinations.

### 2.5. Evaluation of Mitochondrial ROS Production in Leukocytes

Mitochondrial ROS production was evaluated by static cytometry using a fluorescence microscope (IX81; Olympus, Hamburg, Germany) coupled with the static cytometry software ScanR (Olympus, Hamburg, Germany). After extraction, fresh PMNs were seeded in 48-well plates (at 1.5 × 105 PMNs per well) and incubated for 30 min with red mitochondrial superoxide indicator (MitoSOX, 5 μM). Cells from each patient were seeded in triplicate, and 12 images per well were recorded. To visualize nuclei, we coupled both fluorochromes with Hoechst 33342 (4 μM, Sigma Aldrich, St. Louis, MO, USA). Fluorescence was standardized and referred to as a percentage of control.

### 2.6. Western Blot Analysis

Leukocytes were incubated for 15 min on ice with a lysis buffer (400 mM NaCl, 20 mM HEPES pH 7.5, 0.1 mM EDTA, 20% glycerol, 10 μM Na2MoO4, and 0.5% Nonidet P-40) containing protease inhibitors (10 mM β-glycerolphosphate, 10 mM NaF, 10 mM PNP, and 1 mM Na3VO4) and 1 mM dithiothreitol and were then centrifuged at 4 °C for 15 min. Protein concentrations were determined using the BCA protein assay kit (Thermo Fisher Scientific, Chicago, IL, USA). Protein samples (25 µg) were resolved by means of sodium dodecyl sulfate polyacrylamide gel electrophoresis and then transferred to nitrocellulose membranes. After blocking, they were incubated with primary antibodies overnight at 4 °C. We used the following primary antibodies: anti-NFκB-p65 (phospho S536) rabbit polyclonal antibody (Abcam, Cambridge, MA, USA) and anti-β actin rabbit polyclonal antibody (Sigma Aldrich, MO, USA). Blots were incubated with goat anti-rabbit HRP secondary antibody (Millipore Iberica, Madrid, Spain) and developed for 2 min with supersignal west femto (Thermo Fisher Scientific, IL, USA). Chemiluminescence signals were detected with a Fusion FX5 acquisition system (Vilbert Lourmat, Marne La Vallée, France) and analyzed by densitometry using Bio1D software (Vilbert Lourmat, Marne La Vallée, France). Protein bands were normalized to the expression of β-actin in the same sample.

### 2.7. Gene Expression Analysis

RNA was isolated from leukocytes using a GeneAll Ribospin Total RNA extraction kit (GeneAll Biotechnology, Hilden, Germany) following the manufacturer’s indications. RNA quantification was obtained using a NanoDrop 200c spectrophotometer (Life Technologies, Thermo Fisher Scientific), and purity was confirmed with the 260 nm/280 nm and 260 nm/230 nm absorbance ratios. cDNA was generated with the RevertAid first-strand cDNA synthesis kit (Life Technologies, Thermo Fisher Scientific). Working aliquots (1:10 *v*/*v*) of the first-strand cDNA were prepared, and 2 µL of these aliquots was used in further steps. We assessed NFkB-p65(RelA) relative gene expression using quantitative RT-PCR in a 7500 Fast RT-PCR system (Life Technologies, Carlsbad, CA, USA) and the 2−ΔΔCT method. β-actin gene expression was employed as an endogenous control, and we calculated the average ∆Ct of the basal group to calculate the ∆∆Ct values for every sample. qRT-PCR reactions were carried out as follows: 10 min at 95 °C, 40 cycles (designed in one step) at 95 °C for 10 s, and one cycle at 60 °C for 30 s, as well as a melting curve stage. For the reaction mix, we used LightCycler^®^ 480 SYBR Green I Master (Roche, Mannheim, Germany). Data were analyzed with Expression Suite software (Life Technologies, Thermo Fisher Scientific) and Microsoft Excel. The specific sequence, accession number, and annealing temperature of each primer are shown in Table 1.

### 2.8. Statistical Analysis

Statistical analysis was carried out using GraphPad Prism version 7.00 (GraphPad Software, La Jolla, CA, USA, www.graphpad.com, accessed 15 January 2021). Normality was confirmed using the Shapiro–Wilk test. Parametric data were expressed as mean ± standard deviation (SD), and non-parametric data as a median with 25th and 75th percentiles. Statistical significance between groups was assessed by one-way ANOVA followed by a Tukey or Dunnett multiple comparisons test for parametric data, or the Friedman test followed by Dunn’s multiple comparisons test for non-parametric data. A paired or unpaired *t*-test was employed when two groups were compared. Differences of *p* < 0.05 were considered statistically significant. Bar graphs show mean ± standard error of the mean (SEM).

## 3. Results

### 3.1. Anthropometric and Biochemical Analysis

This study initially involved eighteen T2D patients who initiated treatment with empagliflozin. All patients had received stable glucose-lowering therapy for at least 12 months before being recruited for the study, and they continued with this therapy in combination with empagliflozin during the entire study period. The information compiled regarding the concomitant medications taken by the participants in our study is summarized in Table 2.

In terms of the medical history of microvascular complications, four and five diabetic patients were affected by retinopathy and nephropathy, respectively.

Table 3 shows the anthropometric and biochemical data for our study population. We observed that empagliflozin significantly reduced the body weight of the participants at 12 weeks of treatment (*p* < 0.01) and that this reduction was maintained at 24 weeks (*p* < 0.01 vs. baseline and *p* < 0.05 vs. 12 weeks). In parallel, a significant waist circumference reduction was observed at 24 weeks (*p* < 0.01 vs. baseline and *p* < 0.05 vs. 12 weeks). These data were confirmed by a progressive reduction in BMI (*p* < 0.05 at 12 weeks and *p* < 0.01 at 24 weeks, both vs. baseline). The data show a decrease in glucose levels after 24 weeks (*p* < 0.05) and in HbA1C levels from 12 weeks onwards (*p* < 0.05).

Total cholesterol levels were increased at 12 weeks (*p* < 0.05) and were maintained at 24 weeks (*p* < 0.05). No significant changes in LDL-c and HDL-c were observed after treatment with empagliflozin. Patients receiving insulin as part of their treatment were excluded from HOMA-IR and insulin assessments. Lastly, we did not observe differences in triglyceride and hs-CRP levels.

### 3.2. Leukocyte–Endothelium Interactions and Adhesion Molecule Expression

**Figure 1:**
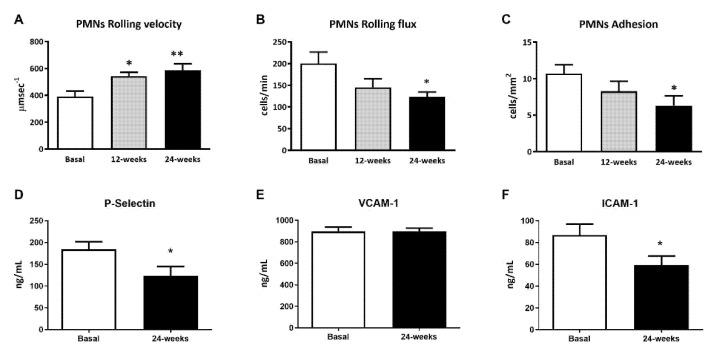
Effects of empagliflozin treatment on leukocyte–endothelium interactions and serum soluble cell adhesion molecules in type 2 diabetic patients at baseline and at 12 and 24 weeks of treatment. (**A**) Leukocyte rolling velocity (µm·sec^−1^), (**B**) rolling flux (cells/min), (**C**) leukocyte adhesion (cells/mm^2^), (**D**) P-Selectin levels (ng/mL), (**E**) VCAM-1 levels (ng/mL), (**F**) ICAM-1 levels (ng/mL). For leukocyte–endothelium interactions, an ANOVA followed by Dunnett’s multiple comparisons test was performed. For serum soluble cell adhesion molecules, Student’s t-test was performed. * *p* < 0.05 and ** *p* < 0.01 vs. baseline. Data are expressed as mean ± SEM. Abbreviations: PMN, polymorphonuclear leukocytes; ICAM-1, intercellular adhesion molecule-1; P-Selectin, platelet selectin; VCAM-1, vascular cell adhesion molecule-1.

The leukocyte rolling velocity (Figure 1A) was enhanced at 12 and 24 weeks of treatment with empagliflozin (*p* < 0.05 and *p* < 0.01, respectively) compared to baseline. Regarding the PMN rolling flux and adhesion (Figure 1B,C), data for both show a tendency to decrease, which became significant at 24 weeks of treatment (*p* < 0.05 both). To explore, in more depth, the results obtained during the leukocyte–endothelium interaction assays, we studied the expression of adhesion molecules in the serum at 24 weeks of treatment. We observed a significant reduction in P-Selectin and ICAM-1 expression levels at 24 weeks (Figure 1D,F; *p* < 0.05), but not in the expression of VCAM-1 (Figure 1D,E).

### 3.3. Mitochondrial Superoxide Production

**Figure 2:**
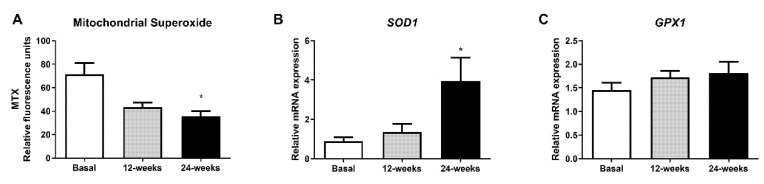
Effects of empagliflozin treatment on mitochondrial superoxide production and expression of *SOD1* and *GPX1* in leukocytes from type 2 diabetic patients at baseline and at 12 and 24 weeks of treatment. (**A**) Mitochondrial superoxide production, (**B**) mRNA expression of *SOD1*, and (**C**) mRNA expression of *GPX1* in human leukocytes. Data are expressed as mean ± SEM. Mitochondrial superoxide was measured as relative MitoSOX fluorescence by static cytometry, and data were normalized with respect to fluorescence at baseline. The values of *SOD1* and *GPX1* gene expression were normalized to mean baseline mRNA expression levels and calculated using the 2−ΔΔCT method. For mitochondrial superoxide production, an ANOVA followed by Dunnett’s multiple comparisons test was performed. For mRNA expression of *SOD1* and *GPX1*, a paired Student’s t-test was performed. * *p* < 0.05 vs baseline. Abbreviations: MTX, MitoSOX Red mitochondrial superoxide indicator; *GPX1*, glutathione peroxidase 1; *SOD1*, superoxide dismutase 1.

To evaluate whether empagliflozin had an effect on oxidative stress parameters, we measured mitochondrial superoxide production. The fluorescence of MitoSOX decreased significantly at 24 weeks of treatment (Figure 2A; *p* < 0.05), indicating a reduction in oxidative stress in leukocytes from T2D patients. Interestingly, this reduction was associated with a significant increase in the mRNA expression of *SOD1* (Figure 2B; *p* < 0.05), and a tendency for *GPX1* to rise after 24 weeks of treatment (Figure 2C).

### 3.4. Inflammatory Parameters

**Figure 3:**
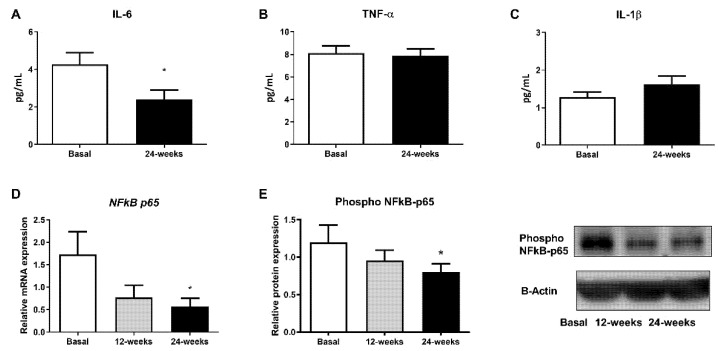
Effects of empagliflozin treatment on serum levels of pro-inflammatory cytokines IL-6, TNF-α, and IL-1β, as well as production and expression of NFκB-p65, in leukocytes from T2D patients at baseline and at 12 and 24 weeks of treatment. Serum levels of (**A**) IL-6, (**B**) TNF-α, and (**C**) IL-1β, (**D**) mRNA expression of NFkB-p65, and (**E**) protein levels of phospho NFκB-p65, representative WB images in human leukocytes. Data are expressed as mean ± SEM. The values of NFkB-p65 gene expression were normalized to mean baseline mRNA expression levels and calculated using the 2−ΔΔCT method. For mRNA expression of NFkB-p65, a paired Student’s t-test was performed. * *p* < 0.05 vs. baseline. Abbreviations: IL, interleukin; NFκB-p65, nuclear factor kappa-light-chain-enhancer of activated B cells; TNF-α, tumor necrosis factor alpha.

Changes in diabetes-related inflammatory status were measured in terms of IL-6, TNF-α, and IL-1β levels in the serum and phospho NFκB-p65 protein expression. Empagliflozin treatment was able to markedly reduce IL-6 serum levels, although no changes in TNF-α and IL-1β were detected (Figure 3A; *p* < 0.05, Figure 3B,C). Moreover, a decrease in phospho NFκB-p65 levels in leukocytes from T2D patients at 24 weeks of empagliflozin treatment (Figure 3E; *p* < 0.05) was observed. In parallel, we obtained similar results after evaluating gene expression (Figure 3D; *p* < 0.05).

## 4. Discussion

In this observational, prospective follow-up study, we analyzed the effects of the iSGLT2 empagliflozin on cardiovascular parameters, the atherosclerotic process, and inflammation, including leukocyte–endothelium interactions, adhesion molecules, mitochondrial ROS, and the serum and gene expression profile of different pro-inflammatory markers after 12 and 24 weeks of treatment. Furthermore, we explored some of the beneficial effects of empagliflozin, including weight reduction, decreased BMI and waist circumference, and improved glucose and HbA1C levels [5].

We evaluated the effects of empagliflozin on leukocyte–endothelial cell interactions by using an ex vivo parallel-flow chamber assay that mimics the physiological blood flow. As a result, we observed that empagliflozin increases the PMN rolling velocity in consonance with a decrease in the PMN rolling flux and adhesion at 24 weeks of treatment. These actions suggest that this drug exerts a beneficial effect by protecting against the early stages of the atherosclerotic process. Hyperglycemia and increased levels of HbA1C are key factors in the atherosclerosis process and are related to enhanced leukocyte–endothelium interactions, mitochondrial impairment, and oxidative stress [8]. Enhanced leukocyte–endothelium interactions have also been linked to insulin resistance [8]. Our data confirm that empagliflozin treatment reduces hyperglycemia in general, which would improve insulin resistance. In line with the beneficial effect of empagliflozin, the drug has been reported to protect the heart from inflammation and energy depletion via AMPK activation, as well as preventing doxorubicin-induced myocardial dysfunction [9].

Of note, an increase in total cholesterol, LDL-c, and HDL-c plasma levels has also been shown in T2D patients treated with empagliflozin, as previously reported [10,11]. The mechanism by which SGLT2 inhibition raises these levels is yet to be determined. It has been suggested that the increase in cholesterol is partly attributable to hemoconcentration, as SGLT2 inhibitors induce volume contraction as a response to the increased urinary volume [12,13]. It is important to note that, although our study participants tended to have higher levels of cholesterol following treatment with empagliflozin, the mean of these concentrations was lower than that of healthy individuals, since the former group had been receiving statins, which are known to block the rate-limiting step of cholesterol synthesis and reduce the relative risk of cardiovascular disease associated with diabetes [14].

In addition, endothelial–leukocyte interactions depend on the levels of adhesion molecules resulting from vascular inflammation and dysfunction and involved in the recruitment of immune cells and platelets to the endothelium. In this sense, the present results show a reduction in the expression of the adhesion molecules P-selectin and ICAM-1 after 24 weeks of empagliflozin treatment. These results are in accordance with those previously reported by our group showing that empagliflozin reduces the levels of the inflammatory enzyme myeloperoxidase—which is actively involved in the development of microvascular alterations and increased release of the anti-inflammatory interleukin-10 (IL-10). Considered as a whole, these data suggest that empagliflozin ameliorates the inflammatory state and reduces the risk of CVDs. In support of this, empagliflozin has been shown to reduce inflammation and boost the antioxidant response of leukocytes from T2D patients [15].

It is well known that T2D is related to oxidative stress and that this leads to pro-inflammatory responses. In fact, enhanced ROS levels activate the pro-inflammatory nuclear factor NFκB, thus contributing to insulin resistance. Considering this, we decided to explore whether empagliflozin can modulate mitochondrial ROS levels as well as NFκB-p65 protein and gene expression in leukocytes from T2D patients. Interestingly, we showed that empagliflozin decreases mitochondrial ROS production. This reduction was associated with a significant increase in the mRNA expression of *SOD1*, and a tendency towards an increased mRNA expression of *GPX1* expression, two critical markers of the mitochondrial antioxidant capacity. Moreover, the expression of p65 (phospho S563) was reduced by the treatment, highlighting empagliflozin as a molecule with anti-inflammatory properties that, by decreasing IL-6 levels, modulates not only oxidative stress and leukocyte–endothelium interactions but also the inflammatory response.

## 5. Conclusions

In conclusion, this preliminary study provides evidence that treatment with empagliflozin decreases leukocyte–endothelium interactions, adhesion molecules, mitochondrial ROS, and IL-6 and NFkB expression in T2D and enhances antioxidant activity. This highlights the value of this drug for preventing the atherosclerotic process, inflammation, and, consequently, possible cardiovascular events in T2D patients.

## 6. Study’s Limitations

This study has some limitations. The potential effects of empagliflozin are based on observational and prospective studies with a limited number of patients. However, we performed this pilot study with the primary aim of estimating average values and variability in order to plan future studies in larger populations. In this sense, although the sample size is quite limited, this study provides valuable preliminary evidence of the anti-inflammatory and antioxidant properties of empagliflozin treatment in humans, properties which may underlie its beneficial cardiovascular effects.

## Figures and Tables

**Table 1:** Primer sequences used in qRT-PCR assays.

Gene Name	Primer Name	Primer Sequence	Sequence Accession Number
*Superoxide dismutase 1*	*SOD1* Forward	GGTGTGGCCGATGTGTCTAT	NM_000454
	*SOD1* Reverse	TTCCACCTTTGCCCAAGTCA	
*Glutathione peroxidase 1*	*GPX1* Forward	TTGAGAAGTTCCTGGTGGGC	NM_000581.4
	*GPX1* Reverse	CGATGTCAGGCTCGATGTCA	
*RELA proto-oncogene*, *NF-kB subunit*	*NFkB p65* Forward	ATCCCATCTTTGACAATCGTGC	NM_021975
	*NFkB p65* Reverse	CTGGTCCCGTGAAATACACCTC	
*Actin beta*	*Actin B* Forward	CCTCGCCTTTGCCGATCC	NM_001101
	*Actin B* Reverse	CGCGGCGATATCATCATCC	

**Table 2:** List of concomitant medications.

Patient	Lipid-Lowering Medication	Antihypertensive Medication	Antithrombotic Medication	Diuretic Medication
1	-	-	-	-
2	Atorvastatin	-	-	-
3	Excluded from the study			
4	Atorvastatin	-	ASA	-
5	Atorvastatin	Enalapril + bimatoprost + timolol	-	HCTZ
6	Simvastatin	Valsartan + Amlodipine	-	HCTZ
7	-	-	-	-
8	Atorvastatin	Ramipril	ASA	-
9	Atorvastatin	Nebivolol + Valsartan	Clopidogrel + ASA	-
10	Pravastatin + Fenofibrate	-	-	-
11	Atorvastatin	-	ASA	-
12	-	-	-	-
13	Simvastatin	Manidipine + Olmesartan	-	HCTZ
14	Atorvastatin	Amlodipine + Irbesartan	-	HCTZ
15	Atorvastatin	Eprosartan		HCTZ
16	Simvastatin	Telmisartan	ASA	HCTZ
17	Atorvastatin	-	-	-
18	Excluded from the study			

Abbreviations: ASA, acetylsalicylic acid; HCTZ, hydrochlorothiazide.

**Table 3:** Anthropometric characteristics of the study population at baseline, and at 12- and 24-week follow-up.

	Baseline	12-Week Empagliflozin	24-Week Empagliflozin
N	16	16	16
Age (years)	59.7 ± 10.8	-	-
Sex (female)	5		
Weight (kg)	85.7 ± 20.1	82.9 ± 20.3 **	81.6 ± 20.3 ** ^#^
Waist circumference (cm)	102.7 ± 12.3	99.8 ± 13.5	97.1 ± 13.7 ** ^#^
BMI	31.4 ± 5.3	30.3 ± 5.4 *	29.9 ± 5.6 **
SBP (mmHg)	139.5 ± 26.9	139.6 ± 24.6	133.9 ± 21.7
DBP (mmHg)	76.4 ± 14.1	81.0 ± 16.3	73.9 ± 12.7
Glucose (mg/dL)	149.1 ± 35.9	134.1 ± 32.6	125.2 ± 19.9 *
HbA_1C_ (%)	7.6 ± 1.3	7.2 ± 1.3 *	6.8 ± 0.9 *
Insulin (μUI/mL)	9.6 ± 5.4	9.6 ± 5.5	9.5 ± 5.9
HOMA-IR	3.88 ± 2.16	3.32 ± 1.54	3.10 ± 2.10
Total cholesterol (mg/dL)	141.0 ± 25.4	154.7 ± 27.6 *	149.5 ± 27.1 *
LDL-c (mg/dL)	82.3 ± 16.9	87.2 ± 16.6	89.1 ± 19.8
HDL-c (mg/dL)	46.1 ± 6.1	43.2 ± 7.1	47.5 ± 3.7
Triglycerides (mg/dL)	92 (83–131)	113 (100–168)	104 (82–122)
hs-RCP	2.42 (1.2–11.5)	4.28 (1.5–7.8)	1.9 (1.3–5.8)

Data are expressed as mean ± SD for parametric variables and median (interquartile range) for non-parametric data. The following statistical analyses were performed: for parametric variables, a repeated measures one-way ANOVA followed by Tukey’s multiple comparisons test; for non-parametric variables, a Friedman test followed by Dunn’s multiple comparisons test. * *p* < 0.05 vs. baseline, ** *p* < 0.01 vs. baseline, ^#^ *p* < 0.05 vs. 12-week empagliflozin treatment. Abbreviations: BMI, body mass index; DBP, diastolic blood pressure; HbA1C, glycated hemoglobin A1C; HDL-c, high-density lipoprotein cholesterol; HOMA-IR, homeostasis model assessment of insulin resistance; hs-CRP, high-sensitivity C-reactive protein; LDL-c, low-density lipoprotein cholesterol; SBP, systolic blood pressure.

## Data Availability

The datasets used and/or analyzed during the current study are available from the corresponding author on reasonable request. The data are not publicly available due to patient’s privacy.
